# Rheology of Gum Arabic Polymer and Gum Arabic Coated Nanoparticle for enhanced recovery of Nigerian medium crude oil under varying temperatures

**DOI:** 10.1016/j.dib.2018.06.075

**Published:** 2018-06-26

**Authors:** Oyinkepreye D. Orodu, Kale B. Orodu, Richard O. Afolabi, Eboh A. Dafe

**Affiliations:** Department of Petroleum Engineering, Covenant University, P.M.B 1023 Ota, Ogun State, Nigeria

**Keywords:** Oil recovery, Temperature effect, Gum Arabic, Nanoparticles, Nigerian medium crude oil

## Abstract

The dataset in this article are related to the rheology of dispersions containing Gum Arabic coated Alumina Nanoparticles (GCNPs) and Gum Arabic (GA) polymer for Enhanced Oil Recovery (EOR) of Nigerian medium crude oil under varying temperatures. The data included the viscosity of the dispersion containing GCNPs compared to GA at different shear rates. In addition, data on the rheological properties (plastic viscosity, yield point, and apparent viscosity) of the dispersions under varying temperatures was also presented.

**Specifications Table**

**Table:** 

Subject area	*Petroleum Engineering*
More specific subject area	*Enhanced Oil Recovery/Tertiary Oil Recovery*
Type of Data	*Tables and Figures*
How Data was Acquired	*Rheological Study using the OFITE®Model 800 Viscometer*
Data Format	*Raw Data*
Experimental Factors	*Varying Temperatures*
Experimental Features	*1.* *GCNP preparation using Al_2_O_3_ nanoparticles and Gum Arabic* *2.* *A rheological study using OFITE^®^ Model 800 Viscometer at different temperatures for GA polymer and GCNPs*
Data Source Location	*Department of Petroleum Engineering, Covenant University, Nigeria*
Data Accessibility	*Data is with the article*

**Value of data**

• The data shows the relevance of polymer coated nanoparticles for the recovery of crude oil from conventional reservoirs under high-temperature conditions. The underlying mechanism in the thermal behavior of the GCNPs is required in explaining the trend in viscosity transition from 100 to 150 °C as captured in Fig. 1(a-c).

**Fig. 1:**
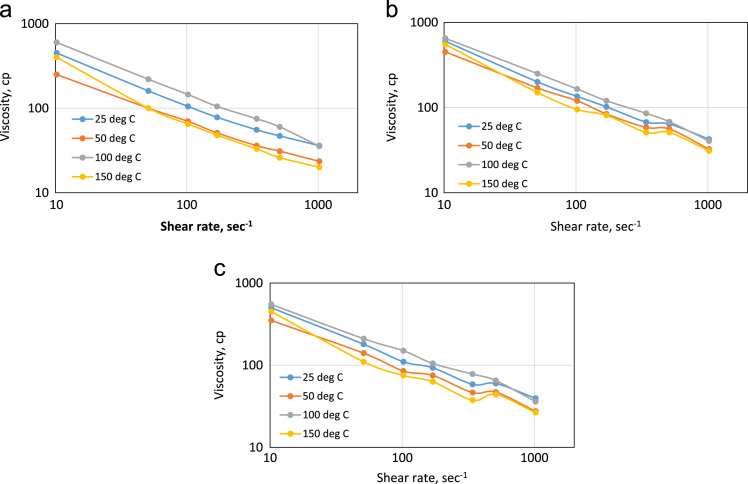
Viscosity profile for (a) GA polymer solution (b) GCNPs (1.33 wt% GA-5 wt% Nanoparticles) (c) GCNPs (1.33 wt% GA-5wt% nanoparticles.
• The data presented also shows that the quantity of nanoparticles in the GCNPs have a direct impact on the viscous property for the recovery of oil beyond the capacity of GA polymer flooding under high-temperature conditions (Table 2 and Fig. 2). This further indicates that a detailed characterization of the structural state and impact on nanoparticle content on the rheological performance of GCNPs is required.

**Fig. 2:**
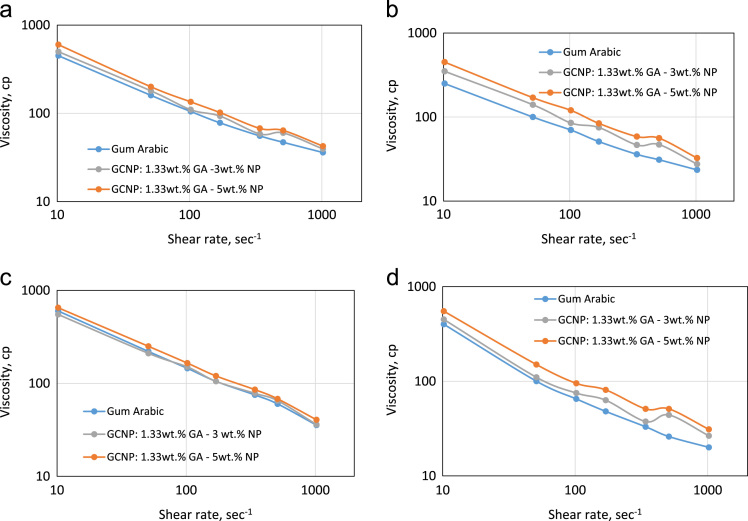
Viscosity profile (a) 25 °C (b) 50 °C (c) 100 °C and (d) 150 °C.
• The results obtained calls for a detailed study of the mechanisms at play with respect to the polymeric and surfactant property of Gum Arabic. Likewise, the performance of Gum Arabic should be evaluated and compared to that of known and standard polymers used in the industry.

## 1 Data

Nanoparticles are reported in [1], [2], [3] to improve oil recovery but its instability paved the way for stable polymer-coated nanoparticles [4], [5]. The dataset presented in this paper provides an experimental investigation on temperature effect on the rheology of Gum Arabic (GA) polymer and Gum Arabic Coated Nanoparticle (GCNPs) for Enhanced Recovery of Nigerian Medium Crude Oil. Gum Arabic is a naturally occurring polymer that is abundant in Nigeria and Sudan. Table 1 shows the dataset on the rheological properties of Gum Arabic Polymer, GCNPs (1.33 wt% GA - 5 wt% NP, GCNPs) and GCNPs (1.33 wt% GA - 3 wt% NP, GCNPs) at varying temperatures and shear rates. Table 2 shows the viscosity data values for GA and GCNPs at given temperatures of 25, 50, 100 and 150 °C. Whereas Fig. 1 displays graphically, Viscosity profile for (a) GA polymer solution (b) GCNPs (1.33 wt% GA-5wt% Nanoparticles) (c) GCNPs (1.33 wt% GA-5wt% Nanoparticles). Fig. 2 displays the viscosity profile of GA polymer and GCNPs at 25, 50, 100 and 150 °C.

**Table 1:** Rheology of Gum Arabic Polymer, GCNPs (1.33 wt% GA - 5 wt% NP, GCNPs) and GCNPs (1.33 wt% GA - 3 wt% NP, GCNPs).

**Shear rate (1/s)**	**Temperature °C**			
	25	50	100	150
	**Viscosity (cp)**			
**Rheology of Gum Arabic Polymer**				
1021.38	36	23.5	35.5	20
510.69	47	31	60	26
340.46	55.5	36	75	33
170.23	78	51	105	48
102.138	105	70	145	65
51.069	160	100	220	100
10.2138	450	250	600	400
**Plastic Viscosity (cp)**	25	16	11	14
**Yield Point (lb/100 ft2)**	22	15	49	12
**Apparent Viscosity (cp)**	36	23.5	35.5	20

**Rheology of (1.33 wt% GA - 5 wt% NP, GCNPs)**				
1021.38	42.5	32.5	40.5	31
510.69	64	56	68	51
340.46	67.5	58.5	85.5	51
170.23	102	84	120	81
102.138	135	120	165	95
51.069	200	170	250	150
10.2138	600	450	650	550
**Plastic Viscosity (cp)**	21	9	13	11
**Yield Point (lb/100 ft2)**	43	47	55	40
**Apparent Viscosity (cp)**	42.5	32.5	40.5	31

**Rheology of (1.33 wt% GA - 3 wt% NP, GCNPs)**				
1021.38	39.5	27.5	36	26.5
510.69	60	47	65	44
340.46	58.5	46.5	78	37.5
170.23	93	75	105	63
102.138	110	85	150	75
51.069	180	140	210	110
10.2138	500	350	550	450
**Plastic Viscosity (cp)**	19	8	7	9
**Yield Point (lb/100 ft2)**	41	39	58	35
**Apparent Viscosity (cp)**	39.5	27.5	36	26.5

**Table 2:** Viscosity values for GA and GCNPs at given temperatures of 25, 50, 100 and 150 °C.

**Shear rate (1/s)**	GA	GCNP: 1.33–3 wt%	GCNP: 1.33 wt%- 5 wt%
**Rheology @ 25 °C**			
1021.38	36	42.5	39.5
510.69	47	64	60
340.46	55.5	67.5	58.5
170.23	78	102	93
102.138	105	135	110
51.069	160	200	180
10.2138	450	600	500
**Rheology @ 50 °C**			
1021.38	23.5	32.5	27.5
510.69	31	56	47
340.46	36	58.5	46.5
170.23	51	84	75
102.138	70	120	85
51.069	100	170	140
10.2138	250	450	350
**Rheology @ 100 °C**			
1021.38	35.5	40.5	36
510.69	60	68	65
340.46	75	85.5	78
170.23	105	120	105
102.138	145	165	150
51.069	220	250	210
10.2138	600	650	550

**Rheology @ 150 **°**C**			
1021.38	20	31	26.5
510.69	26	51	44
340.46	33	51	37.5
170.23	48	81	63
102.138	65	95	75
51.069	100	150	110
10.2138	400	550	450

## 2 Experimental design, materials and methods

### 2.1 Preparation of Gum Arabic Coated Nanoparticles (GCNPs)

The nanoparticle in use was Al_2_O_3_ (30–60 nm, purity greater than 99%; manufactured by Sigma Aldrich and purchased from Equilab Solutions in Nigeria.). 50 g of Al_2_O_3_ was dispersed in 1 l of deionized water to make nano-fluid suspensions, making a 5 wt% mixture. It was further diluted to 3 wt% in order to completely carry out further experiments. The Gum Arabic (a polymer; purchased locally in Nigeria) was mixed with the prepared nanofluids at a concentration of 1.33 wt. %. Finally, the concentration of GCNPs prepared are 1.33 wt% Gum Arabic – 3.0 wt% Al_2_O_3_ Nanoparticles –GCNPs and 1.33 wt% Gum Arabic – 5.0 wt% Al_2_O_3_ Nanoparticles – GCNPs.

### 2.2 Rheological analysis of Gum Arabic (GA) and Gum Arabic Coated Nanoparticles (GCNPs)

The OFITE Model 800 (8-Speed) Viscometer was used to obtain dial readings, *θ*, at various RPM (Revolution per Minute) values (3, 6, 30, 60, 100, 200, 300, and 600). Based on the API specification 13A, the Eqs. (1), (2), (3) was used in estimating the rheological properties of the prepared drilling mud.

(1) $$\mathrm{Plastic}\;\mathrm{Viscosity}\;\left(\mathrm{PV}\right),\mathrm{cp}\;=\;\theta_{600}\;-\;\theta_{300}$$

(2) $$\mathrm{Yield}\;\mathrm{Point}\;\left(\mathrm{YP}\right),\frac{\mathrm{Ib}}{100{\mathrm{ft}}^{2}}\;=\;\theta_{300}\;-\;\mathrm{PV}$$

(3) $$\mathrm{Apparent}\;\mathrm{Viscosity}\;\left(\mathrm{AV}\right),\mathrm{cp}\;=\;\theta_{600}/2$$

*θ*_600_ and *θ*_300_ are the dial readings at 600 and 300 RPM respectively. The viscosity values were calculated using the relationship in Eq. (4) as specified in the OFITE operational manual (1cp is equivalent to $10^{-3}\mathrm{Pas}$).

(4) $$\eta =\mathrm{KF}\frac{\theta }{\mathrm{RPM}}$$

η is the viscosity in cp, *K* is the machine constant of the Rotor – Bob combination (R1B1) = 300, *F* is the spring factor = 1 for the R1B1 combination.
